# Co-occurrence of yeast, streptococci, dental decay, and gingivitis in the post-partum period: results of a longitudinal study

**DOI:** 10.1080/20002297.2020.1746494

**Published:** 2020-04-15

**Authors:** Kirtana Ramadugu, Freida Blostein, Deesha Bhaumik, Wenwen Jiang, Elyse Davis, Elizabeth Salzman, Usha Srinivasan, Carl F. Marrs, Katherine Neiswanger, Daniel W. McNeil, Mary L. Marazita, Betsy Foxman

**Affiliations:** a Center for Molecular and Clinical Epidemiology of Infectious Diseases, University of Michigan School of Public Health Department of Epidemiology, Ann Arbor, MI, USA; b Center for Craniofacial and Dental Genetics, Department of Oral Biology, School of Dental Medicine, University of Pittsburgh, Pittsburgh, PA, USA; c Center for Oral Health Research in Appalachia, University of Pittsburgh, Pittsburgh, Pennsylvania, USA, and West Virginia University, Morgantown, West Virginia, USA; d Departments of Psychology and Dental Practice & Rural Health, West Virginia University, Morgantown, WV, USA; e Department of Human Genetics, Graduate School of Public Health; and Clinical and Translational Sciences Institute, School of Medicine, University of Pittsburgh, Pittsburgh, PA, USA

**Keywords:** Dental caries, gingivitis, salivary microbiome, mitis streptococci

## Abstract

**Objective**: The interactions between yeast and streptococci species that lead to dental decay and gingivitis are poorly understood. Our study describes these associations among a cohort of 101 post-partum women enrolled in the Center for Oral Health Research in Appalachia, 2012–2013.

**Methods**: All eligible women without dental caries were included (n = 21) and the remainder were randomly sampled to represent the total number of decayed, missing, and filled teeth (DMFT) at enrollment. We used amplicon sequencing and qPCR of saliva from 2, 6, 12 and 24 visits to determine microbiome composition.

**Results**: Active decay and generalized gingivitis were strongly predictive of each other. Using adjusted marginal models, *Candida albicans* and *Streptococcus mutans* combined were associated with active decay (OR = 3.13; 95% CI 1.26, 7.75). However, *C. albicans* alone (OR = 2.33; 95% CI: 0.81, 6.75) was associated with generalized gingivitis, but *S. mutans* alone was not (OR = 0.55; 95% CI: 0.21, 1.44). Models including microbiome community state types (CSTs) showed CSTs positively associated with active decay were negatively associated with generalized gingivitis.

**Discussion**: *C. albicans* is associated with active decay and generalized gingivitis, but whether one or both are present depends on the structure of the co-existing microbial community.

## Background

The impact of dental disease is significant. An analysis of the United States 2011–2014 National Health and Nutrition Examination Survey estimated that 12.5 million children and 57.6 million adults have untreated dental caries [1]. In 2015, the estimated per person expenditures due to dental diseases were 340.47, USD with total expenditures exceeding 119 USD billion. Lost productivity in the USA due to decay of deciduous teeth was estimated at more than 223 USD million, and for permanent teeth, almost 5 USD billion [2].

Dental decay results when tooth enamel demineralization outpaces remineralization [3–5]. Demineralization is primarily caused by acidogenic bacteria, which feed on carbohydrates and produce weak organic acids [3–5]. Acidogenic bacteria do not live in isolation: they operate and interact in complex, dynamic, multispecies biofilms, and interactions within biofilms may differentially affect caries risk [6]. Understanding the mechanisms through which microbes interact with each other and other host and environmental factors can provide novel interventions to prevent caries formation.

Human cariogenic bacteria *Streptococcus mutans* and *Streptococcus sobrinus* have high cariogenic potential as they both produce and tolerate acid [4,5]. Other streptococci such as *Streptococcus oralis* and *Streptococcus mitis* contribute less to acidification but may enable enhanced colonization of tooth surfaces by other microbes [7]. Streptococci and candida (yeast) are common in the oral cavity and interact within multi-species biofilms [8]. *C. albicans* and *S. mutans* are frequently detected together in oral biofilms [9]; both are associated with early childhood caries [7]. Studies *in vitro* have demonstrated that *Candida albicans* can promote the ability of streptococci to form biofilms on surfaces [10,11], and that extracellular factors derived from the association between *C. albicans* and *S. mutans* may stimulate *S. mutans* growth [12]. Further, *S. oralis* can induce changes in *C. albicans* which promote coaggregation and mucosal biofilm growth [13]. The complexity of microbiome interactions may explain the weak predictive power of *S. mutans* presence for caries when considered alone [14]. Moreover, inflammation of the gums (gingivitis) often follows the accumulation of dental plaque; thus, interactions between *C. albicans* and Streptococcal species leading to biofilm development could lead to gingivitis. Studies of the co-occurrence of yeast and streptococci species in humans are needed to help resolve these issues.

Pregnancy and the early post-partum period are thought to be high-risk periods for oral disease. During pregnancy, women are at increased risk of gingivitis, dental caries, and other oral conditions [15]. Few studies have examined changes in active decay during the partum and post-partum period; however, risk factors for caries progression such as salivary pH and the buffering effect of saliva against pH acidification are recorded to decrease during the last trimester of pregnancy and recover soon after delivery [16,17]. In a study of 16 pregnant women, Laine *et al*. found an increase in salivary *S. mutans*, yeast, and lactobacilli during the third trimester of pregnancy and during lactation, with decreasing counts post-lactation. Subgingival counts of *Eubacterium, Fusobacterium, Prevotella, Staphylococcus,* and *Streptococcus* species were elevated during the 12th week of pregnancy compared to 4–6 weeks post-partum [16,17]. The post-partum period is also of interest as *S. mutans* may be transmitted vertically from mother to child; thus controlling maternal levels of *S. mutans* may help intervene on childhood caries formation [18].

This cohort study examines the role of co-occurrence of *C. albicans* and selected Streptococcal species on dental caries and generalized gingivitis among post-partum mothers while taking into account behavioral, medical, and microbial factors. Specifically, we tested the following hypotheses (a) there are shifts in oral microbiota throughout the post-partum period, (b) associations among selected Streptococcal species and *C. albicans* lead to active decay and gingivitis, and (c) behavioral, medical, and microbial factors act together to cause active decay and gingivitis.

## Material and methods

### Study population

Women were recruited into the COHRA (Center for Oral Health Research in Appalachia, cohort 2) in Pittsburgh, Pennsylvania, and multiple sites in West Virginia during their pregnancies. Eligibility criteria included (i) women over the age of 17 who were between 12 and 29 weeks pregnant at the time of enrollment, (ii) women without tuberculosis or who were not immunocompromised, (iii) women who stayed in Pennsylvania or West Virginia for the duration of the study, (iv) infants who were not born prematurely (>35 weeks) and did not have any serious medical conditions, and (v) Caucasian. Participants were followed longitudinally for the first 2 years of infant life.

### Data collection

A trained, calibrated dentist or dental hygienist assessed the mother’s oral health and collected oral specimens from mothers at each in-person visit. Former training/calibration sessions were conducted on a regular basis for examiners from all sites. Inter-rater reliability scores (Cohen’s *kappa*) for sound, decayed, and filled groups surface codes ranged from 82.1 to 92.5. Specific protocol for the clinical examination and calibration is described elsewhere [19]. Gingivitis was classified based on a soft tissue examination into localized, generalized, or hyperplasia. For this study, we only considered generalized gingivitis. Saliva samples were collected through spitting and stored using the OMNIgene Discover kit (DNA Genotek).

Surveys were administered to gather information on maternal demographic variables (education, employment, and income), behavioral variables (smoking, alcohol consumption), breast or formula-feeding habits, antibiotic usage, maternal and infant oral health status, and delivery mode during in-person visits at the COHRA recruitment sites, plus telephone interviews conducted by the University of Pittsburgh Center for Social and Urban Research (UCSUR). Informed consent was gained from all participating adults and parental guardians for each child. The study protocol was reviewed and approved by the University of Pittsburgh and West Virginia University institutional review boards.

### Selection of study sample

We selected for this analysis a sample of 101 mothers who donated saliva at the 2-month and 12-month post-partum visits for Pennsylvania participants and the 2-month and 24-month post-partum visits for West Virginia participants, all of whom had a 2-month visit between 2012 and 2013. Two samples from the 12-month visit for West Virginia were also included (Figure S1). To assess the impact of pregnancy and behavioral factors on the mother’s oral health, all eligible women without dental caries prior to giving birth were sampled (n = 21) and the remaining 80 women were randomly sampled from a list ordered by the number of decayed, missing, or filled teeth (DMFT) to represent the caries distribution at enrollment. Healthy mothers were defined as having a DMFT score of 0 at the 2-month visit; the presence of white spots was not considered carious.

### DNA extraction

Salivary DNA was extracted using DNeasy® Blood & Tissue kits and the automated QIAcube benchtop apparatus, both products of QIAGEN (Venlo, Netherlands). Eighty microlitre of enzyme cocktail and 100ul of sample were incubated at 37°C for 1 h. The enzyme cocktail was comprised of 22.5:4.5:1.125:1.125:1 parts cell lysis solution (Promega, Madison, WI, USA): lysozyme: mutanolysin: RNaseA: lysostaphin (Sigma-Aldrich, St. Louis, MO, USA), respectively. Incubation was followed by DNA extraction in the QIAcube and measurement of DNA concentration using a Nanodrop 2000 C spectrophotometer (Thermo Scientific, Waltham, MA, USA). Samples were stored at −80°C.

### qPCR for specific species

DNA samples were examined by quantitative PCR (qPCR) for *Streptococcus mitis, S. sobrinus, S. mutans, S. oralis*, and *Candida albicans* using protocols and conserved primers described elsewhere [20–22]. The total reaction volume was 10 ul, consisting of 5:2.5:2:0.5 parts SYBR green PCR master mix (Sso EvaGreen Supermix, Bio-Rad, Hercules, CA): DNA template: nuclease-free water: forward and reverse primer pair at a 5 uM concentration, respectively. Primer sequences and protocols used are described in Supplemental Table 1. Standard curves for individual plates were normalized to adjust for potential technician bias. The limit of detection was 100 genomic copies/ml; all samples with abundance values below this threshold were considered negative. Assays were run in duplicate, with a positive and negative control in each run. Discordant results were re-tested. Mean abundance values for each sample-species were calculated.

**Table 1.** Sociodemographic characteristics of selected sample^a^ of Center for Oral Health Research in Appalachia Cohort 2 at the 2-month visit by study site

	Pennsylvania (N = 77) No. (%)	West Virginia (N = 24) No. (%)	*P* Value
Mother’s Age:			0.02
18–24	10 (13.0%)	9 (37.5%)	
25–34	54 (70.1%)	10 (41.7%)	
35+	13 (16.9%)	5 (20.8%)	
Delivery Route:			0.88
C-section	22 (28.6%)	8 (33.3%)	
Missing	2 (2.6%)	0 (0.0%)	
Vaginal	53 (68.8%)	16 (66.7%)	
Household Income:			0.10
Missing	2 (2.6%)	1 (4.2%)	
Under $25,000	18 (23.4%)	8 (33.3%)	
Between $25,000 and $50,000	13 (16.9%)	8 (33.3%)	
Between $50,000 and $100,000	28 (36.4%)	6 (25.0%)	
Over $100,000	16 (20.8%)	1 (4.2%)	
Education Status:			0.03
High school or less	13 (16.9%)	9 (37.5%)	
Associate’s degree or less	21 (27.3%)	8 (33.3%)	
Bachelor’s degree	27 (35.1%)	4 (16.7%)	
Graduate school degree	16 (20.8%)	2 (8.3%)	
Missing	0 (0.0%)	1 (4.2%)	

p-values compare visits within sites. ^a^Sampled from COHRA 2 participants enrolled between 2012 and 2013. Women with no decayed missing or filled teeth (DMFT = 0) were oversampled (n = 21); the remaining participants were selected by ordering by DMFT and taking a systematic sample to represent the range of DMFT among participants enrolled by 2013.

### Amplicon sequencing for characterization of salivary microbiome composition

The V6 region of the 16s rRNA gene was amplified in the sample set, using primers and protocols described elsewhere [23,24]. The forward and reverse primer sequences mapped to the 967–985 (CAACGC-GARGAACCTTACC) and 1078–1061 (ACAACACGAGCTGACGAC) on the *Escherichia coli* 16s rRNA segment, with unique 8-nucleotide error-correcting barcodes added to the reverse primer for each sample. Each PCR reaction contained 25 ng of sample DNA, 2.5 ul 10 uM forward primer, 2.5 ul 10 uM reverse primer plus barcode, 25 ul Promega’s GoTaq® Green Master Mix, and enough PCR-grade water so that the final reaction volume was 50 ul. The PCR mixture was amplified in an S1000™ thermocycler (Bio-Rad, Hercules, CA, USA), using the protocol: 25 cycles at 94°C for 0:45 S, 5°C for 0:45 S, and 72°C for 0:45 S. After amplification, 6 ul of PCR product was run on a 1.5% gel at 100 V to visualize the presence of a band close to the 130 base-pair region. Successfully amplified PCR products were purified, re-quantified using the Nanodrop 2000 C and stored at −20°C. Any sample with a DNA concentration of less than 5 ng/ul for two measurements was re-amplified. If the problem persisted following re-amplification, DNA from that respective sample was re-extracted.

Three nanograms of DNA from each sample’s successfully amplified and the purified PCR product was pooled into a single 1.5 ml Eppendorf tube. An additional 140 ng of known mock community DNA was added to the pool as a quality control measure for sequencing. The pool was concentrated to 55 ng/uL using Amicon® centrifugal filter units for benchtop centrifuges (Millipore, Billerica, MA, USA) and split into 2 equal volumes. The two technical replicates were sequenced on separate lanes of an Illumina HiSeq platform with 100 paired-end cycles at the University of Michigan Sequencing Core.

### Bioinformatics

The 143,046,307 raw sequences were processed and analyzed with a university-wide Flux high-performance computing cluster. Paired ends were joined using FLASH; sequences were quality-filtered and de-multiplexed with the split_libraries.py script in QIIME 1.9.0. This resulted in 98,582,469 sequences with an average length of 124 base pairs (bp). The reads were then clustered into oligotypes using an unsupervised minimum entropy decomposition (MED) method described elsewhere [25]. Briefly, MED uses Shannon entropy to identify regions of the sequence that are information-rich and iteratively partitions the sequence dataset into nodes until there is no remaining entropy [25]. The minimum substantive abundance of an oligotype (-M) criterion was set to 19,706, and the maximum variation allowed in each node (-V) criterion was set to 1. Of the total 98,582,469 reads, MED removed 21,724,727 reads due to the – M and – V criteria and clustered the remaining 76,803,742 reads into 366 oligotypes (average of 180,551 reads per sample). Taxonomy for each oligotype was assigned to the CORE reference database using the UCLUST consensus taxonomy assigner in QIIME 1.9.0. Ninety-seven per cent were assigned at genus level and 72% at species level. CORE is a specialized database for the identification of bacteria within the oral microbiome [26].

### Community state type generation

Community state types (CSTs), or groups of communities with similar microbial composition, were defined using Dirichlet multinomial mixture models [27]. A tab-delimited text file with sample counts of oligotypes for both mother and infant data was collapsed into the most abundant genera; this served as input into these models. The number of CSTs in the population was determined by selecting the least amount of Dirichlet components that provided the lowest Laplace approximation of model fit, in our case 4 CSTs [27]. CST generation was conducted using the DirichletMultinomial package in R.

Each sample was assigned to the CST with the greatest posterior probability determined by the Dirichlet model. Misclassification criteria included: (i) samples with a maximum probability score of less than 80% for the most probable community type or (ii) samples that had a greater than 10% probability of being classified as any other community type. All samples in this dataset were classified using this criterion.

### Statistical analysis

Participant demographics were assessed using frequency counts, proportions, and standard *t*-tests or chi-square tests. The abundance of each streptococci species was log-transformed, and the average mean was compared across categories of infant’s age post-partum, study site, and presence or absence of active caries; this was then evaluated using repeated measure one-way ANOVA tests or chi-squared/Wilcoxon tests.

To account for participants where values for critical variables were missing, we used hierarchical multiple imputation [28,29]. As a sensitivity analysis, we excluded those with missing values; this did not change the findings from results using the imputed values. With multiple imputations, a number of replacements for the missing data are drawn from the distribution of the missing values, given the observed data and an imputation model. The following variables were imputed using the jomo package in R: smoking behavior, alcohol consumption, breastfeeding behavior and presence of *S. mutans, S. mitis, S. sobrinus, S. oralis, C. albicans*, and generalized gingivitis. There were 16 participants with missing values for *S. mutans, S. mitis, S. sobrinus, S. oralis*, or *C. albicans.*

Multivariate marginal logistic regression models using the imputed data were used to identify associations between *C. albicans, S. mutans,* and active decay. Smoking behavior, alcohol consumption, breastfeeding behavior, site, and generalized gingivitis were included as covariates. To take into account repeated measures (each participant is included twice) we used an exchangeable covariance structure. Because CSTs derived from 16 S rRNA taxonomic screens are correlated with qPCR results, additional marginal models with exchangeable covariance structures and the same additional covariates were run using CST, rather than qPCR results, as the main exposure of interest. We also fit marginals models using generalized gingivitis as the outcome, with smoking behavior, alcohol consumption, breastfeeding behavior, site, and active decay as covariates.

To further explore the associations of specific taxa with active decay and generalized gingivitis, we compared the relative abundance of taxa by decay status and gingivitis using ALDEx2 [30]. ALDEx2 uses the centered log-ratio (clr) transformation that ensures the data are scale invariant and sub-compositionally coherent. We used Benjamini and Hochberg [31] to correct for multiple hypothesis testing setting the false discovery rate to 0.1 and a p < 0.01. The effect size in ALDEx2 measures whether the difference within each group is greater than the difference between groups standardized for the variation between the groups [32].

## Results

At the 2-month post-partum visit, women from Pennsylvania (n = 77) and West Virginia (n = 24) were similar with respect to age and delivery mode. However, women in Pennsylvania had higher incomes and greater levels of formal education (Table 1). Pennsylvania women were slightly more likely than West Virginia women to be breastfeeding their infants at 2 months (62.3% versus 54.2%) and were less likely than West Virginia women to report smoking at 2-month post-partum but more likely to report drinking alcohol (Table 2). At 2 months, Pennsylvania mothers averaged one tooth with active decay (1.28; sd: 3.17; range 0 to 14) and had an average DMFT of 6.9 (sd: 6.84; range 0 to 28); West Virginia mothers averaged 2.38 teeth (sd: 3.25; range 0 to 11) and had an average DMFT of 8.6 (sd: 6.50; range 0 to 24). There was little change in DMFT between the 2 months and 12 months (Pennsylvania) or 24 months (West Virginia) follow-up visits, but more active decay was detected at follow-up among women from West Virginia compared to Pennsylvania. In both groups, the percent with generalized gingivitis increased between the two visits.

**Table 2.** Oral health and health behaviors of selected sample^a^ of Center for Oral Health Research in Appalachia (COHRA) Cohort 2 at 2-month visit by study site

	Pennsylvania			West Virginia^b^		
	~ 2-month visit (N = 77)	~12-month visit (N = 77)		~ 2-month visit (N = 24)	~24-month visit (N = 22)	
	No. (%)	No. (%)	*P* value	No. (%)	No. (%)	*P* value
Active Decay Detected	21 (27.3%)	26 (33.8%)	0.48	13 (54.2%)	16 (72.7%)	0.34
Currently Breastfeeding	48 (62.3%)	25 (32.5%)	<0.001	13 (54.2%)	1 (4.55%)	<0.001
Currently Smoking	19 (24.7%)	22 (28.9%)	0.68	10 (43.5%)	11 (50.0%)	0.53
Currently Drinking Alcohol	46 (59.7%)	62 (81.6%)	0.005	3 (13.0%)	10 (45.5%)	0.03
Decayed, Missing and Filled Tooth (DMFT)			0.82			0.97
DMFT 1–7	29 (37.7%)	31 (40.3%)		12 (50.0%)	9 (40.9%)	
DMFT 8+	32 (41.6%)	33 (42.9%)		10 (41.7%)	11 (50.0%)	
No Caries	16 (20.8%)	13 (16.9%)		2 (8.3%)	2 (9.1%)	
Generalized gingivitis	10 (13.0%)	15 (19.5%)	0.38	5 (20.8%)	11 (50.0%)	0.07
Localized gingivitis	49 (63.6%)	42 (54.5%)	0.33	6 (25.0%)	9 (40.9%)	0.35

p-values compare visits within sites.

^a^Sampled from COHRA 2 participants with a 2-month visit between 2012 and 2013. Women with no decayed missing or filled teeth (DMFT = 0) were oversampled (n = 21); the remaining participants were selected by ordering by DMFT and taking a systematic sample to represent the range of DMFT among participants with a 2-month visit by 2013.

^b^In addition, we included a 12-month visit for two West Virginia participants. One of these participants had a DMFT of 1–7 the other a DMFT of 8+; one had generalized gingivitis and the other active decay.

### Relationship between community state types, active decay, and generalized gingivitis

**Figure 1.**
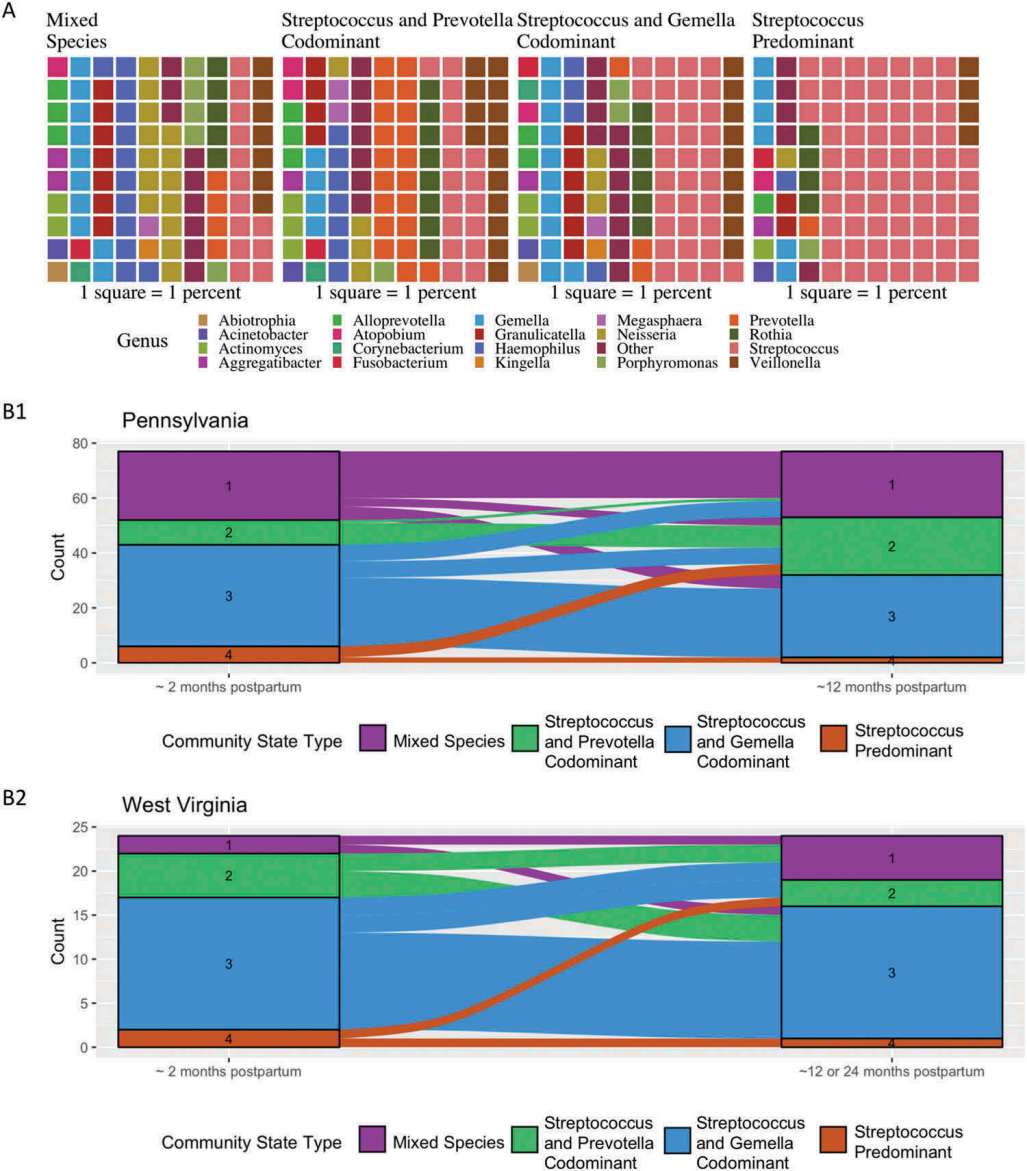
(a) Waffle plots of the average relative abundance for the four community state types (CSTs) at the genus level. Each square represents a one percent contribution to the average relative abundance. (b1) Dynamics of CST between 2-months (n = 77) and 12-months (n = 75) post-partum (Pennsylvania participants). (b2). Dynamics of CST between 2 months (n = 24) and 12 (n = 2) or 24 (n = 22) months post-partum (West Virginia participants). Women participating in the Center for Oral Health Research in Appalachia Cohort 2 from Pennsylvania and West Virginia

**Figure 2.**
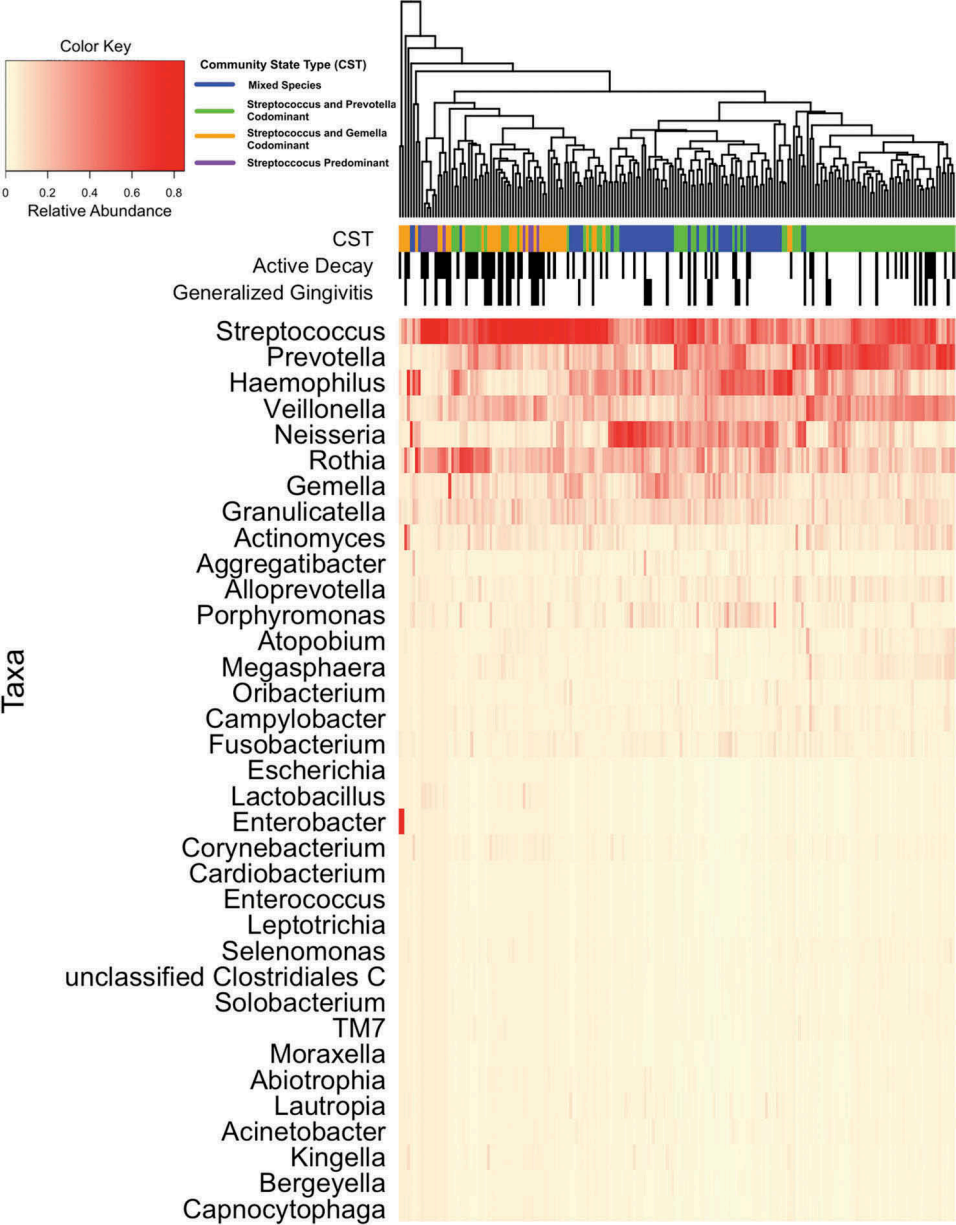
Heat map showing the relative abundance of salivary taxa at 2 months and 12 months (n = 77) post-partum (Pennsylvania participants) and 2 and 12 (n = 2) or 2 and 24 (n = 22) months post-partum (West Virginian participants). Women participating in the Center for Oral Health Research in Appalachia Cohort 2. Each column represents results from one sample; each individual appears twice. Dendrogram shows Bray-Curtis distance between salivary microbiomes by the overall distribution of taxa. The top three rows under the dendrogram indicate community state type (CST), presence of active decay, and generalized gingivitis of each individual, respectively. CSTs were identified using Dirichlet multinomial mixed models and named based on the organisms present

Using Dirichlet multinomial mixture models, we identified four CSTs in the salivary microbiome: (1) Mixed Species (average Shannon diversity 3.99); (2) *Streptococcus* and *Prevotella* codominant (average Shannon diversity 4.01); (3) *Streptococcus* and *Gemella* codominant (average Shannon diversity 3.60); and, (4) *Streptococcus* predominant (average Shannon diversity 2.96) (Figure 1(a)). The majority of women stayed within the same CST between the two time points (52/77 (68%) in Pennsylvania and 13/24 (54%) in West Virginia) (Figure 1(b1,b2)). We next grouped salivary microbiomes by Bray-Curtis distance, and displayed on a heat map, indicating CST, presence of active decay, and generalized gingivitis (Figure 2). No clear patterns were observed between CST and active decay or generalized gingivitis or between microbiomes that were more similar and presence of active decay and generalized gingivitis.

### Prevalence of selected microbes detected using qPCR

**Figure 3.**
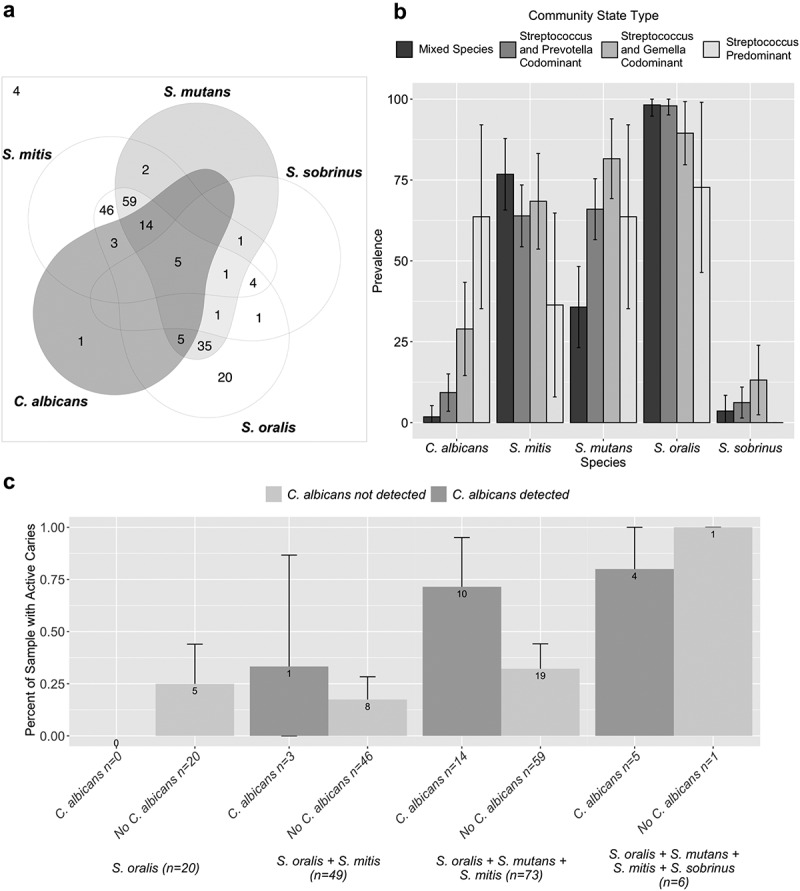
Colonization with mitis streptococci and *Candida albicans* and association with dental decay including samples from all included visits (n = 202). (a) Venn diagram showing the overlap in colonization among selected mitis streptococci and *C. albicans. C. albicans* mainly occurs in the presence of *S. mutans*. (b) Prevalence of selected mitis streptococci and *C. albicans*, by community state type. (c) Association with active decay by the presence of *C. albicans*. Prevalence of active decay increases in the presence of *C. albicans*. Women participating in the Center for Oral Health Research in Appalachia Cohort 2 from Pennsylvania (n = 77) and West Virginia (n = 24)

The prevalence of *C. albicans, S. mutans, S. mitis, S. oralis,* and *S. sobrinus* detected using qPCR was similar by study site and between visits. To explore the extent that women were colonized by several of these microbes, we used a Venn diagram (Figure 3(a)). The prevalence of *S. mitis, S. oralis,* and *S. sobrinus* overlapped considerably with the prevalence of *S. mutans*; virtually all samples positive for *C. albicans* and *S. mitis, S. oralis,* or *S. sobrinus* also had *S. mutans. C. albicans* was detected significantly more frequently in women who also had *S. mutans* or *S. sobrinus* than in women who did not (Fisher’s Exact test p = 0.003 and p = 0.013, respectively). When *C. albicans* was present in combination with *S. mutans, S. mitis, S. oralis,* or *S. sobrinus*, there was a higher prevalence of active decay, even though the sample sizes are small (Figure 3(c)). *C. albicans* was also detected more frequently in the *Streptococcus* predominant and *Streptococcus* and *Gemella* codominant CSTs and *S. mutans* was detected more frequently in *Streptococcus* and *Gemella* codominant, *Streptococcus* predominant and *Streptococcus* and *Prevotella* codominant than in the mixed-species CST (Figure 3(b)).

### Associations between selected microbes, community state types, active decay, and gingivitis

We used two separate adjusted marginal models to predict the presence of active dental decay: one using qPCR data and one using CSTs. The individual effects of *S. mutans* (OR = 1.84; 95%CI: 0.83, 4.1) and *C. albicans* (OR = 1.47; 95%CI: 0.66, 3.28) suggest an association with decay, and the joint estimate suggests the effects are additive (OR = 2.72; 95%CI: 1.12, 6.62) (Table 3). This was true using only complete observations and also when using all observations with imputed missing values. Similarly, in marginal models using 16SrRNA derived CSTs, compared to mixed-species CST, the *Streptococcus* and *Gemella* codominant CST was significantly associated with active decay (OR = 2.89; 95% CI: 1.08, 7.70), consistent with descriptive findings of a positive association between *Streptococcus* and *Gemella* codominant CST and *S. mutans* and *C. albicans*. In both models, current breastfeeding was negatively associated, and current smoking positively associated with active decay.

**Table 3.** Marginal models predicting active dental decay in 101 Appalachian mothers at 2 points during the post-partum period^a^

	Models including all 185 participants with complete information	Models including all 202 participants (missing values are imputed)
qPCR Detection Models		
	OR (95% CI)	OR (95% CI)
S. *mutans* detected	1.84 (0.83, 4.1)	2.04 (0.94, 4.41)
C. *albicans* detected	1.47 (0.66, 3.28)	1.53 (0.7, 3.38)
Currently drinking alcohol	0.58 (0.3, 1.13)	0.55 (0.29, 1.05)
Generalized gingivitis	4.18 (1.79, 9.75)	4.31 (1.91, 9.72)
West Virginia	1.91 (0.7, 5.19)	2.05 (0.75, 5.56)
Currently Breastfeeding	0.49 (0.27, 0.88)	0.41 (0.22, 0.75)
Currently Smoking	3.59 (1.62, 7.98)	4.00 (1.79, 8.93)
Both S. *mutans* and C. *albicans* detected	2.72 (1.12, 6.62)	3.13 (1.26, 7.75)
Community State Type (CST) Models		
	OR (95% CI)	OR (95% CI)
CST (reference: Mixed Species)		
*Streptococcus* and *Gemella* Codominant	2.89 (1.08, 7.7)	2.97 (1.11, 7.97)
*Streptococcus* and *Prevotella* Codominant	1.94 (0.75, 5.03)	2.16 (0.82, 5.64)
*Streptococcus* Predominant	1.16 (0.1, 12.8)	2.01 (0.26, 15.59)
C. *albicans* detected	1.69 (0.65, 4.39)	1.70 (0.67, 4.31)
Currently drinking alcohol	0.58 (0.31, 1.08)	0.53 (0.29, 0.98)
Generalized gingivitis	4.52 (1.78, 11.46)	5.13 (2.03, 12.95)
West Virginia	2.16 (0.8, 5.86)	2.25 (0.83, 6.14)
Currently Breastfeeding	0.48 (0.26, 0.89)	0.41 (0.22, 0.77)
Currently Smoking	3.07 (1.32, 7.11)	3.18 (1.34, 7.52)

^a^Participants in the Center for Oral Health Research in Appalachia Cohort 2: 77 Pennsylvania women who donated saliva at the 2-month and 12-month visits and 24 West Virginia women who donated saliva at the 2-month and 12 or 24-month post-partum visit.

In the marginal models predicting active decay, there was a strong positive association between generalized gingivitis and active decay. Therefore, we fit an additional set of marginal models predicting the presence of generalized gingivitis, using the same co-variates but substituting active decay for generalized gingivitis. There was also a strong positive association between active decay and gingivitis; however, the association with *S. mutans* (OR = 0.55; 95% CI: 0.21, 1.44) was in the opposite direction from the model of active decay (Table 4). This was also true for the model using CST, where, relative to the mixed-species CST, all CSTs had odds ratios less than 1.0. However, in both these models, *C. albicans* alone had a greater than twofold association with generalized gingivitis. As we observed with active decay, current breastfeeding was negatively associated, and current smoking positively associated with generalized gingivitis.

**Table 4.** Marginal models predicting generalized gingivitis in 101 Appalachian mothers at 2 time points during the post-partum period^a^

	Models including all 185 participants with complete information	Models including all 202 participants (missing values are imputed)
qPCR Detection Models		
	OR (95% CI)	OR (95% CI)
S. *mutans* detected	0.57 (0.2, 1.65)	0.55 (0.21, 1.44)
C. *albicans* detected	2.41 (0.8, 7.24)	2.33 (0.81, 6.75)
Currently drinking alcohol	0.66 (0.27, 1.62)	0.64 (0.28, 1.49)
Active Decay	5.78 (1.91, 17.53)	4.94 (1.63, 14.98)
West Virginia	2.03 (0.62, 6.68)	2.06 (0.67, 6.27)
Currently Breastfeeding	0.15 (0.05, 0.46)	0.21 (0.07, 0.61)
Currently Smoking	1.78 (0.69, 4.61)	1.74 (0.7, 4.34)
Both S. *mutans* and C. *albicans* detected	1.38 (0.44, 4.29)	1.27 (0.41, 3.93)
Community State Type (CST) Models		
	OR (95% CI)	OR (95% CI)
CST (reference: Mixed Species)		
*Streptococcus* and *Gemella* Codominant	0.22 (0.04, 1.12)	0.23 (0.05, 0.97)
*Streptococcus* and *Prevotella* Codominant	0.39 (0.1, 1.55)	0.38 (0.11, 1.33)
*Streptococcus* Predominant	0.17 (0.01, 2.15)	0.16 (0.02, 1.36)
C. *albicans* detected	3.01 (0.96, 9.45)	2.76 (0.88, 8.71)
Currently drinking alcohol	0.58 (0.25, 1.36)	0.54 (0.24, 1.17)
Active Decay	7.69 (2.54, 23.31)	6.63 (2.35, 18.73)
West Virginia	1.87 (0.56, 6.2)	1.75 (0.56, 5.53)
Currently Breastfeeding	0.15 (0.04, 0.52)	0.2 (0.06, 0.63)
Currently Smoking	2.25 (0.77, 6.57)	2.29 (0.81, 6.47)

^a^Participants in the Center for Oral Health Research in Appalachia Cohort 2: 77 Pennsylvania women who donated saliva at the 2-month and 12-month visits and 24 West Virginia women who donated saliva at the 2-month and 12 or 24-month post-partum visit.

### Taxa associated with active decay and generalized gingivitis

To better understand the strong associations between active decay and generalized gingivitis, we used ALDEx2 to compare the relative abundance of taxa among those with both active decay and generalized gingivitis, active decay alone, and those with neither active decay nor generalized gingivitis. Using a false discovery rate of 0.1, microbiome-wide associations between those with active decay or active decay and generalized gingivitis compared to those with neither at 2-month (Figure S2) and 12-month (Figure S3) post-partum show several taxa positively and negatively associated with the presence of active decay and generalized gingivitis after correction for multiple testing. For factors positively associated with active decay and gingivitis at 2 months, effect sizes ranged from −1.25 to −.69 and for those negatively associated from 0.63 to 1.08. For factors positively associated with active decay and gingivitis at 12 months, effect sizes ranged from −0.87 to −0.70 and for those negatively associated from 0.59 to 0.89 (Table 5). Associations were consistent in direction – although no statistical significance – in the two time periods. No taxa at 2 months were associated with active decay alone using a false discovery rate of 0.1 and a p < 0.01 at either time period.

**Table 5.** Results of ALDEx2 analysis comparing women with the combination of active decay and generalized gingivitis to those with neither*. Center for Oral Health Research in Appalachia Cohort 2 participants. Effect sizes of taxa with p values <0.02 at 2 or 12 months; those meeting this criterion are bolded. Negative values represent factors positively associated with active decay and gingivitis; positive values represent a negative association with active decay and gingivitis

	Effect Size	
Taxa	2 months	12 months
*Corynebacterium matruchotii*	**1.019**	0.384
*Fusobacterium nucleatum subsp. fusiforme*	0.600	**0.769**
*Fusobacterium nucleatum subsp. polymorphum*	**−0.826**	−0.477
*Haemophilus parainfluenzae*	**−1.253**	**−0.808**
*Haemophilus_18674*	**−0.687**	−0.647
*Haemophilus_19524*	**−0.941**	**−0.701**
*Haemophilus_nbw161b08c1*	**−1.043**	**−0.749**
*Lactobacillus fermentum*	0.473	**0.588**
*Neisseria meningitidis polysaccharea*	**−0.906**	**−0.835**
*Oribacterium sinus*	**−0.765**	**−0.872**
*Prevotella pleuritidis*	0.891	**0.898**
*Prevotella nigrescens*	**0.891**	0.686
*Prevotella_oral taxon 299*	0.595	−0.834
*Selenomonas noxia*	**0.962**	**0.700**
*Streptococcus peroris*	0.481	**0.666**
*Streptococcus_18830*	**0.740**	**0.767**
*Streptococcus_18832*	0.733	**0.724**
*Streptococcus_18846*	**0.772**	**0.739**
*Streptococcus_6815*	**0.711**	**0.689**
*Streptococcus_GU045364*	**0.734**	**0.765**
*unclassified Clostridiales C sulci infirmum*	**−0.892**	−0.771
*Veillonella atypica dispar parvula*	**0.631**	−0.167
*Veillonella_HB016*	**1.085**	0.104

*12 with active decay and generalized gingivitis and 64 with neither at 2-month post-partum; 15 with active decay and generalized gingivitis and 51 with neither at 12-month post-partum.

Note: Taxa not identified to the species level are given an arbitrary number. These sequences were further identified using NCBI BLAST in Supplemental Table 2.

## Discussion

We describe associations between the presence of active decay and the oral microbial community at two post-partum time points, and by geographic area and behavioral factors among 101 women at high risk of dental decay. The composition of the microbiome overall and of specific taxa was relatively stable between 2 months and 1- or 2-year post-partum and were similar between women enrolled in Pennsylvania and West Virginia. We confirm previously reported associations between the presence of *C. albicans* and active decay, and present data suggesting that there is a higher prevalence of active decay when *C. albicans* and *S. mutans* are both present than in the presence of *S. mutans* alone, consistent with an earlier report among a pediatric population [7]. We further observe that the prevalence of generalized gingivitis increased during the post-partum period, possibly because habits that ceased or lessened during pregnancy – most notably smoking – increased (In Pennsylvania, at 2-month post-partum 24.7% reported smoking compared to 28.6% at 12 months; in West Virginia, at 2-month post-partum 41.7% reported smoking compared to 50% at 24 months). Current breastfeeding was negatively associated with both active decay and generalized gingivitis. Whether this is a marker for physiologic or behavioral changes associated with breastfeeding (or both), or a general focus on health behaviors, is uncertain. Finally, we provide new insights into the known association between gingivitis and dental caries based on assessments of the salivary microbiome.

While many studies *in vitro* and using *in vivo* animal models have reported associations between *C. albicans* and active decay [10,11,33], few have examined the associations with the presence of *S. mutans*. Among 46 caries-free and 51 caries-active 3-to-13-year-old Greek children, prevalence of the combination of *C. albicans* and *S. mutans* in saliva was seven times higher among caries-active children (11/33) compared to caries-free children (2/30) (OR = 7.5; 95% CI 1.51, 37.3) [7]. This effect is larger than reported here; possibly because our study used adults, had a larger sample size, detected the presence of *C. albicans* and *S. mutans* using qPCR rather than culture, and/or adjusted for behavioral variables. We found no studies that assessed the salivary microbiome and *C. albicans* with respect to tooth decay. Although we found a possible association, it might be explained by the higher prevalence in the associated CST of both *C. albicans* and *S. mutans*. Our findings of higher abundance of *Haemophilus parainfluenzae* in the salivary microbiome among participants without active decay or gingivitis compared to those with both are consistent with results of a Danish case-control study of 79 cases with a high level of caries to 85 controls [34].

There are few studies describing the associations between the oral microbiome and gingivitis during the post-partum period. A Singapore longitudinal study characterized the salivary microbiome during each trimester of pregnancy and at 6-week post-partum among 24 women [35]. They observed gingival bleeding increased during the first to the second trimester and decreased towards the post-partum period. Gingival bleeding was associated with *Prevotella-intermedia* and *Porphyromonas endodontalis*, and species identified a keystone for gingivitis also did not overlap with those observed here. Notably, the DMFT was not reported. This makes it difficult to compare to our results as we followed women beginning at 2-month post-partum, and virtually all of our study participants with generalized gingivitis also had active decay. However, an experimental human gingivitis model [36] found *Neisseria spp*. to be negatively associated with gingivitis and *Fusobacterium spp*. to be positively associated with gingivitis in dental plaque. This is consistent with our findings from the salivary microbiome, when we compared those with generalized gingivitis and active decay with participants with neither.

Our study has many strengths. Notably, this is one of the very few studies of oral health during the post-partum period, and ours is the only one reporting longitudinal results from the early post-partum period to one-two years past birth. Participants were examined by calibrated dental professionals, and extensive metadata are available. Further, our population was at high risk of dental decay, increasing our ability to detect associations. Our findings may be somewhat limited because by measuring the salivary microbiome we obtained only an indirect measure of microbial activity leading to dental decay – saliva yields fewer taxa associated with decay than plaque [37]. However, others have reported differences in salivary microbiomes by the presence of decay [38]. Further, we sequenced the V6 region of the 16SrRNA, which provides somewhat less resolution to the species level than some other variable regions. However, resulting decreases in discrimination should lead to an underestimation of differences between groups; i.e. a conservative result. Missing data were handled through multiple imputations. While missing data could be addressed by restricting the analyses to comletely observed cases, this approach is more likely to suffer from low power and give biased results [29].

In conclusion, our study proves new insights into the overlapping etiologies of dental decay and generalized gingivitis. Our results suggest that after taking into account behavioral factors, *C. albicans* is associated with dental decay *and* generalized gingivitis in the immediate post-partum period, but whether one or both are present depends on the structure of the surrounding microbial community.

## Supplementary Material

Supplemental Material

## Data deposition

Raw sequencing data are deposited in an NCBI Sequence Read repository (accession number PRJNA612517).
