# Supplementary material for: Co-occurrence of yeast, streptococci, dental decay, and gingivitis in the post-partum period: results of a longitudinal study
Source: J Oral Microbiol. 2020 Apr 15;12(1):1746494. doi: 10.1080/20002297.2020.1746494 (PMC7178893; doi:10.1080/20002297.2020.1746494)
Supplement: Supplemental Material [file ZJOM_A_1746494_SM1382.zip › supplementary/figure legend.docx]

**Supplemental Figure 1.** Flow chart of study participants, and selected samples during the post-partum period. Women participating the in Center for Oral Health Research in Appalachia Cohort II from Pennsylvania (n=77) and West Virginia (n=24).

**Supplemental Figure 2.** Microbiome wide associations with active decay and the combination of active decay and generalized gingivitis relative to those with neither active decay nor generalized gingivitis at 2 months post-partum. The x axis shows the statistical significance of the association of each taxa (shown on the y axis) with the outcome compared to neither active decay or gingivitis. Points to the right of the vertical line have p values less than 0.1. Effect sizes for each taxa were calculated using ALDEx2, was corrected for multiple testing, and used a false discovery rate of 0.1, meaning that less than 10% of the p values < 0.1 are false positives. Women participating the in Center for Oral Health Research in Appalachia Cohort II from Pennsylvania (n=77) and West Virginia (n = 24).

**Supplemental Figure 3**. Microbiome wide associations with active decay and the combination of active decay and generalized gingivitis relative to those with neither active decay nor generalized gingivitis at 12 months post-partum. The x axis shows the statistical significance of the association of each taxa (shown on the y axis) with the outcome compared to neither active decay or gingivitis. Points to the right of the vertical line have p values less than 0.1. Effect sizes for each taxa were calculated using ALDEx2, was corrected for multiple testing, and used a false discovery rate of 0.1, meaning that less than 10% of the p values < 0.1 are false positives. Women participating the in Center for Oral Health Research in Appalachia Cohort II from Pennsylvania (n=75) and West Virginia (n = 2).
