# Supplementary material for: Co-occurrence of yeast, streptococci, dental decay, and gingivitis in the post-partum period: results of a longitudinal study
Source: J Oral Microbiol. 2020 Apr 15;12(1):1746494. doi: 10.1080/20002297.2020.1746494 (PMC7178893; doi:10.1080/20002297.2020.1746494)
Supplement: Supplemental Material [file ZJOM_A_1746494_SM1382.zip › supplementary/supplementary table.docx]

| Supplemental Table 1. Primer sequences and protocol used for qPCR of saliva DNA samples | | | | |
| --- | --- | --- | --- | --- |
| Organism | Primer Sequence | Target | Protocol | Reference |
| *S. oralis* | Forward 5’-TCCCGGTCAGCAAACTCCAGCC-3’ | *gtfR* | 95 ̊C for 2 min; 40 cycles of 95 ̊C for 15 s, 60 ̊C for 1 min | Henne et al. 2014 [22] |
|  | Reverse 5’-GCAACCTTTGGATTTGCAAC-3′ | *gdh* |  |  |
| *S. mitis* | Forward 5'-TGAAATCGAGGTTGGCCTAC-3’ |  | 95 ̊C for 2 min; 40 cycles of 95 ̊C for 15 s, 60 ̊C for 1 min | Henne et al. 2014 [22] |
|  | Reverse 5'-CGTTTAGGAAAATCTC(G/T)CCCTT-3' |  |  |  |
| *S. mutans* | Forward 5'-GCCTACAGCTCAGAGATGCTATTCT-3' | *gtfB* | 50 ̊C for 2 min; 40 cycles of 95 ̊C for 10 s, 58 ̊C for 1 min | Yoshida et al. 2003 [21] |
|  | Reverse 5'-GCCATACACCACTCATGAATTGA-3’ |  |  |  |
| *S. sobrinus* | Forward 5'-TTCAAAGCCAAGACCAAGCTAGT-3' | *gtfT* | 50 ̊C for 2 min; 40 cycles of 95 ̊C for 10 s, 58 ̊C for 1 min | Yoshida et al. 2003 [21] |
|  | Reverse 5'-CCAGCCTGAGATTCAGCTTGT-3' |  |  |  |
| *C. albicans* | Forward 5'- GGATCGCTTTGACAATGG-3' | 18s | 98 ̊C for 2 min; 40 cycles of 98 ̊C for 1 s, 62 ̊C for 1 s | Rosenthal et al. 2014 [20] |
|  | Reverse 5'- GCGGGTAGTCCTACCTGATTT -3' |  |  |  |

| Supplemental Table 2. NCBI BLAST identification of sequences of taxa not identified in Table 5 | | | |
| --- | --- | --- | --- |
| Taxa | Percent Identity | Query Coverage | Taxa identified via BLAST |
| *Streptococcus*_18830 | 100% | 95% | *Streptococcus salivarius* |
|  | 100% | 95% | *Streptococcus* lactarius |
| *Streptococcus*_18846 | 100% | 97% | *Streptococcus salivarius* |
|  | 100% | 97% | *Streptococcus* lactarius |
| *Streptococcus*_18832 | 100% | 96% | *Streptococcus salivarius* |
|  | 100% | 96% | *Streptococcus* lactarius |
| *Streptococcus*_6815 | 100% | 97% | *Streptococcus salivarius* |
|  | 100% | 97% | *Streptococcus* lactarius |
