# Supplementary figures and images for: Co-occurrence of yeast, streptococci, dental decay, and gingivitis in the post-partum period: results of a longitudinal study
Source: J Oral Microbiol. 2020 Apr 15;12(1):1746494. doi: 10.1080/20002297.2020.1746494 (PMC7178893; doi:10.1080/20002297.2020.1746494)

Supplemental Figure 1


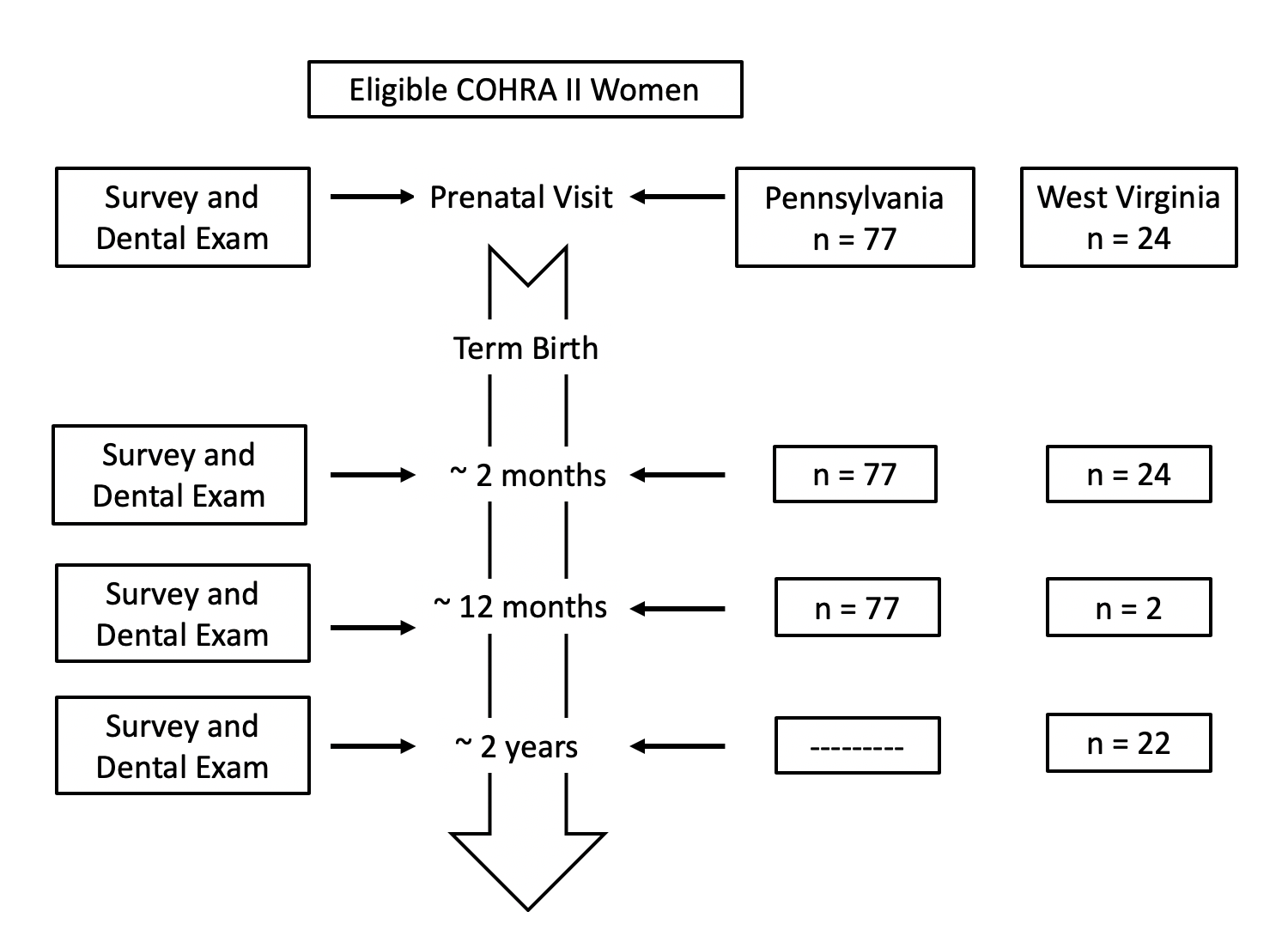


Supplemental Figure 2

Supplemental Figure 3

Supplement: Supplemental Material [file ZJOM_A_1746494_SM1382.zip › supplementary/supplementary figure.docx]
